# Indel detection from DNA and RNA sequencing data with transIndel

**DOI:** 10.1186/s12864-018-4671-4

**Published:** 2018-04-19

**Authors:** Rendong Yang, Jamie L. Van Etten, Scott M. Dehm

**Affiliations:** 1 The Hormel Institute, University of Minnesota, 801 16th AVE NE, Austin, MN 55912 USA; 2 Masonic Cancer Center, University of Minnesota, 420 Delaware St SE, Minneapolis, MN 55455 USA; 3 Department of Laboratory Medicine and Pathology, University of Minnesota, Minneapolis, MN 55455 USA

**Keywords:** Indel detection, RNA-seq, DNA-seq, TCGA, Cancer genome, Exitron, Metastasis

## Abstract

**Background:**

Insertions and deletions (indels) are a major class of genomic variation associated with human disease. Indels are primarily detected from DNA sequencing (DNA-seq) data but their transcriptional consequences remain unexplored due to challenges in discriminating medium-sized and large indels from splicing events in RNA-seq data.

**Results:**

Here, we developed transIndel, a splice-aware algorithm that parses the chimeric alignments predicted by a short read aligner and reconstructs the mid-sized insertions and large deletions based on the linear alignments of split reads from DNA-seq or RNA-seq data. TransIndel exhibits competitive or superior performance over eight state-of-the-art indel detection tools on benchmarks using both synthetic and real DNA-seq data. Additionally, we applied transIndel to DNA-seq and RNA-seq datasets from 333 primary prostate cancer patients from The Cancer Genome Atlas (TCGA) and 59 metastatic prostate cancer patients from AACR-PCF Stand-Up- To-Cancer (SU2C) studies. TransIndel enhanced the taxonomy of DNA- and RNA-level alterations in prostate cancer by identifying recurrent *FOXA1* indels as well as exitron splicing in genes implicated in disease progression.

**Conclusions:**

Our study demonstrates that transIndel is a robust tool for elucidation of medium- and large-sized indels from DNA-seq and RNA-seq data. Including RNA-seq in indel discovery efforts leads to significant improvements in sensitivity for identification of med-sized and large indels missed by DNA-seq, and reveals non-canonical RNA-splicing events in genes associated with disease pathology.

**Electronic supplementary material:**

The online version of this article (10.1186/s12864-018-4671-4) contains supplementary material, which is available to authorized users.

## Background

Advances in DNA-seq and RNA-seq have provided insights into the human genome and transcriptome in health and disease states, including cancer. DNA-seq data is often used as the primary source of mutational information while RNA-seq data is used to measure gene expression. Only in rare instances are DNA-seq and RNA-seq data analyzed together in an integrated fashion. However, there is an increasing recognition that integrated analysis of DNA-seq and RNA-seq data provides a more complete understanding of the molecular genetic state of the cells being studied. For example, integrated analysis of DNA-seq and RNA-seq data could be used to determine whether DNA variants are expressed, identify alterations in genomic DNA when altered DNA fragments escape hybrid capture in whole exome sequencing applications, or identify non-canonical RNA splicing events that are caused by underlying DNA alterations [1].

Although computational tools have been developed to detect single nucleotide variants, small indels (< 10 bp), and structural variations (or gene fusions) from RNA-seq data [2–7], the field currently lacks an effective method to predict indels, especially for mid-sized and large indels, from RNA-seq data. Detecting indels in RNA-seq data is challenging for three reasons. First, RNA-seq aligners fail to map short reads which contain mid-sized insertions and large deletions, as these will be marked as splicing junctions [8]. Second, existing indel callers were developed to predict indels from DNA-seq data, so utilization with RNA-seq data results in extremely high false positive rates of medium- and large-size indel calls due to inability to account for splicing events [8]. Third, it is difficult to distinguish genomic indels from non-canonical splicing events, including microexons and exitrons [9].

To address this critical gap, we developed transIndel, an algorithm that flexibly detects indels from DNA-seq or RNA-seq data. TransIndel parses the chimeric alignments predicted by a short read aligner and reconstructs the mid-sized insertions and large deletions based on the linear alignments of split reads. When analyzing RNA-seq data, transIndel uses several filters to distinguish deletions with RNA splicing events. In this study, we validated the performance of transIndel for detection of small and large indels using simulated DNA-seq data and a 50× whole genome sequencing data set. We applied transIndel to large-scale prostate cancer DNA-seq and RNA-seq data sets and reported novel recurrent *FOXA1* indels and exitron splicing events.

## Implementation

### The transIndel pipeline

TransIndel is intended for paired-end or single-end read data with reads of at least 75 bp. It takes aligned short read data in BAM format as input. The alignment is performed by BWA-MEM [10] for either DNA-seq or RNA-seq as this aligner supports soft clipping at the 5′ or 3′ end of reads and reports chimeric alignments with an ‘SA’ tag in the alignment records. The aligned reads are then passed through a series of processing stages. 1) Preprocessing: the input BAM is parsed and multiple quality filters are applied, including removal of multi-mapped reads (indicated by an “XA” tag in their BAM records), low-quality (MAPQ in the BAM record is less than a user specified cutoff, 60 by default) and secondary alignments (flagged by 0 × 100 in their BAM records). 2) Extracting reads: the chimeric reads are extracted if a) they are labeled with an “SA” tag; b) there is only one alternative hit reported as part of this chimeric alignment and c) mapping quality of this alternative hit exceed a user specified cutoff (60 by default). 3) Classifying linear alignments: each of the two linear alignments in a chimeric alignment is classified into one of the three types based on their CIGAR strings: left part mapped but right part soft clipped, left part soft clipped but right part mapped and others. 4) Indel reconstruction: an indel is detected based on the chromosome, location, strand and type of the two linear alignments in a chimeric alignment and the CIGAR string of this chimeric alignment is redefined as a linear alignment which has included the inferred indel event. Details are described in the section “Indel reconstruction.” After those steps, a new BAM file with a corrected CIGAR string is generated. The indels are detected at nucleotide sites using SAMtools pileup function [11] implemented by a Python module called pysam (http://pysam.readthedocs.io/). When calling the indels at each loci from the newly created BAM file, we only utilize reads with mapping quality score ≥ 15 and require at least 2 reads to support the variant allele. The identified indels are finally reported in VCF format. However, the output BAM file from transIndel can be passed into an existing variant caller such as VarDict [12] for germline or somatic indel calling as well. In our analyses of TCGA data, we used VarDict to estimate the VAF of *FOXA1* indels.

### Indel reconstruction

**Fig. 1:**
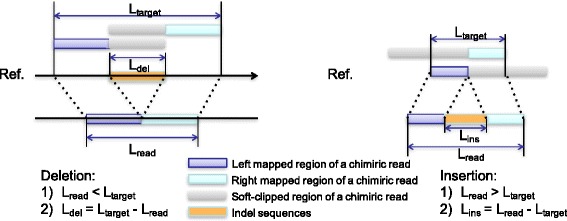
Conceptual overview of transIndel algorithm. Deletions and insertions are recovered from representative and alternative alignments of chimeric reads. Size of the indels are determined based on the difference of target offset and read length

Here we named the types of linear alignments as MS, SM, and O where MS stands for the alignments whose left part mapped but right part soft clipped (e.g. 40M60S as the CIGAR string); SM stands for the alignments whose right part mapped but left part soft clipped (e.g. 40S60M as the CIGAR string) and O stands for the alignments that were not included in the MS or SM class. The indels are inferred based on the genomic location and types of the two linear alignments in a chimeric read as described below and Fig. 1. For the two linear alignments, one is the representative alignment which is reported as the read alignment (denoted by RL) and the other is the alternative alignment which is reported by the SA tag (denoted by AL).

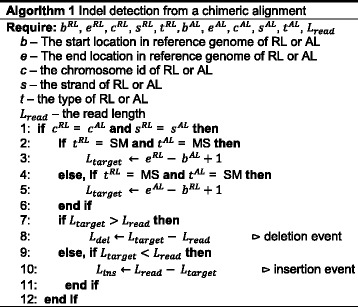



For RNA-seq data, transIndel reports a candidate deletion if they meet the following two criteria: 1) the breakpoints of deletions are not overlapped within a user specified range (default 20 bp) of annotated splice sites defined in a user specified GTF file (in this study, we used the hg19 GTF file from UCSC genome browser Refseq gene track), and 2) the four bases at the breakpoints are not the known splicing motifs (GU-AG, GC-AG and AU-AC).

### Performance comparison with simulated and real data

We compared transIndel’s performance with seven widely used indel detection methods (Pindel v0.2.5, GATK HaplotypeCaller v3.4.46, Platypus v0.8.1, Scalpel v0.4.1, Delly v0.7.6, FermiKit v0.13, and NovoBreak v1.1.3) in both simulated and real DNA sequencing data. Simulated data were generated as described previously [13]. In general, three sequencing coverage are provided at 10×, 20× and 50×. At the 50× coverage, we simulated paired-end reads at four different sizes: 2X50bp, 2X75bp, 2X100bp and 2X200bp. For other coverage depths, we only chose 2X100bp as the read length. The performance of each tool on the simulation data was measured by precision and recall as defined previously [13].

NA12878 WGS raw fastq files were obtained from European Nucleotide Archives with the accession number ERA172924. Paired-end reads were aligned to the GRCh37 human reference using BWA-MEM v0.7.10 with default parameters and then duplicate reads were removed using Picard MarkDuplicates v1.68 (http://broadinstitute.github.io/picard/). We required transIndel to predict the indels in NA12878 with the minimal VAF 0.05 and minimal indel length 1 bp. Default settings were used for all other tools except Scalpel, which was used with –-window 600 when running it for WGS data as recommended by the Scalpel manual (http://scalpel.sourceforge.net/manual.html). The GIAB call set V2.19 was downloaded from NCBI (ftp://ftp-trace.ncbi.nih.gov/giab/ftp/release/NA12878_HG001/NISTv2.19/). GATK was not used when comparing different algorithms to this truth set because it was primarily derived from GATK-based analyses. NovoBreak was not used for NA12878 data set since it is a somatic variant caller. The large deletion reference data set was downloaded from 1000 Genomes Phase 3 structural variation call set (ftp://ftp.1000genomes.ebi.ac.uk/vol1/ftp/phase3/integrated_sv_map/ALL.wgs.mergedSV.v8.20130502.svs.genotypes.vcf.gz). We extracted 1310 deletions detected in NA12878 samples. GATK and Scalpel’s results were not shown as there were zero large deletions called.

The real tumor data set was downloaded from dbGaP (Study ID: phs001223.v1.p1) which included targeted paired-end DNA sequencing data sets from 42 prostate cancer specimens [14]. These specimens had been validated by PCR for multiple genomic rearrangement events, including deletions, duplications, inversions, and translocations in the androgen receptor (AR) gene. We obtained all samples that were validated to harbor deletion events and applied transIndel to those samples for testing with minimal VAF set to 1%.

### Tumor exome and transcriptome data sets and processing

We downloaded tumor and matched normal exome BAM files of 333 TCGA primary prostate adenocarcinoma (PRAD) samples from the Cancer Genomics Hub (CGHub; accessed November 2015). BAM files were converted to FastQ files and realigned to hg19 using BWA-MEM. PCR duplicate reads were removed using Picard MarkDuplicates. The molecular subtypes to which patients had been assigned by the original TCGA study [15] were obtained from cBioPortal (http://www.cbioportal.org/study.do?cancer_study_id=prad_tcga_pub). Somatic indels reported by the original literature were obtained from the Broad Institute FireBrowse portal (http://firebrowse.org/?cohort=PRAD). We obtained the tumor and matched normal exome and tumor poly(A) captured RNA-seq raw FastQ files and somatic indels reported in the original literature of 59 metastatic CRPC specimens from the AACR-PCF SU2C study from dbGap with accession number of phs000915.v1.p1. FastQ files were aligned to hg19 using BWA-MEM and PCR duplicate reads were removed using Picard MarkDuplicates. mRNA expression RPKM values were obtained from cBioPortal (http://www.cbioportal.org/study?id=prad_su2c_2015). The exome and RNA-seq BAM files of the LNCaP cell line were obtained from CGHub (accessed November 2015) under The Cancer Cell Line Encyclopedia (CCLE) project. We converted the BAM files to FastQ files and realigned to hg19 using BWA-MEM followed by duplicated read removal. TransIndel was applied to tumor exome and RNA-seq data to call indels, requiring at least 2 supporting reads with VAF ≥ 10% and minimal indel size ≥10 bp. Overlap of indel calls between DNA-seq and RNA-seq was deemed positive if the detected indels had identical genomic coordinate, type and size of variants. For WES data, indels were called separately from tumor and normal samples using transIndel. Somatic indels were determined with two criteria: 1) a simple subtraction method [16] was applied to remove the indels that were detected in the normal samples or 2) split reads were found mapped to the breakpoints of the tumor exome indels in the matched normal exome BAMs. We limited our analysis to the indels that completely resided within a RefSeq gene. The functional annotations of indels were produced by Ensemble Variant Effect Predictor (http://useast.ensembl.org/Tools/VEP) and UCSC Variant Annotation Integrator [17].

### Compilation of cancer related genes

A total of 2225 candidate cancer related genes were compiled from the literature, publicly available screening panels, and analysis of publicly available data sources (Additional file 1: Dataset S1). This list included 1279 tumor suppressor genes, 147 oncogenes and 799 cancer-associated genes.

### Experimental validation

LNCaP (ATCC, #CRL-1740) cells were obtained from American Type Culture Collection (ATCC). ATCC ensures authenticity of these human cell lines using short tandem repeat analysis. Aliquots of cell culture supernatants from cells in active culture were evaluated regularly for mycoplasma contamination using a PCR-based method as described [18]. All cell line experiments were performed within 2–3 months of resuscitation of frozen cell stocks prepared within 3 passages of receipt from ATCC. Total RNA was purified from LNCaP cells seeded in 6 cm dishes in complete medium. RNA was purified using the Reliaprep RNA Cell Miniprep System (Promega, catalog #Z6011) according to manufacturer instructions. RNA was eluted in nuclease free water and stored at − 80 °C. Genomic DNA was purified with the Nucleospin kit for genomic DNA according to manufacturer instructions (Macherey Nagel catalog #740952.250). The concentration of genomic DNA and total RNA was assessed using a NanoDrop spectrophotometer.

RNA was diluted to 250 ng/μL in nuclease free water. Reverse transcription (RT) was performed on 1 μg total RNA with the GoScript Reverse Transcription System (Promega catalog #A5001). RT was performed with either random hexamers or oligo dT primers. Standard PCR was performed using Taq polymerase with 50 ng input cDNA or genomic DNA and ZBTB18 gene specific primers designed to flank the 112 bp deletion found in LNCaP (: Forward: 5′-agctggaaaaacagtagccagc-3′, Reverse: 5′-catcacaggaagcctctttctcca-3′). PCR products were subjected to agarose gel electrophoresis on a 1.5% agarose gel in 1X TAE buffer.

PCR products were cloned into the pCR-II TOPO vector using the TOPO TA Cloning Kit (Invitrogen catalog #45–0640) for downstream Sanger sequencing. Plasmid DNA was isolated using the IBI High Speed Miniprep kit. Sanger sequencing was performed using the kit manufacturer supplied M13-FWD (− 20) primer, 5’-GTAAAACGAGGGCCAG-3′.

## Results

### Indel detection model

The core component of the transIndel algorithm is the ability to infer large deletions and medium-sized insertions from chimeric alignments. Reads with small indels can be represented as a single linear alignment to the reference genome and hence can be used to detect indels from the direct evidence of the alignment by available indel callers. However, as the indel size increases, short read aligners such as STAR [19] fail to map reads with those indels linearly in a single record. Instead, it detects large deletions as splicing junctions (Additional file 2: Figure S1a). Conversely, a chimera-aware aligner such as BWA-MEM [10] aligns short reads in a chimeric alignment, which consists of two linear alignments with each of the hits marked by soft-clipping in the alignment file that may account for half of the soft-clipped reads (Additional file 2: Figure S1b). By leveraging the alignment details for those chimeric reads, it is possible to reconstruct the linear alignment with mid-sized insertions and large deletions from the initial short read alignment and provide a redefined alignment output for downstream indel detection.

First, transIndel searches chimeric alignments from BAM files generated by BWA-MEM and selects those containing two linear alignments that do not have large overlaps but align on the same chromosome and strand. Second, the type and size of indels are determined by comparing the differences between target offset and read length (Fig. 1). In the case of deletions, the target offset is larger than the read length. In cases of mid-sized novel sequence insertions or tandem duplications, the target offset is shorter than the read length.

### Validation on synthetic and real data

**Fig. 2:**
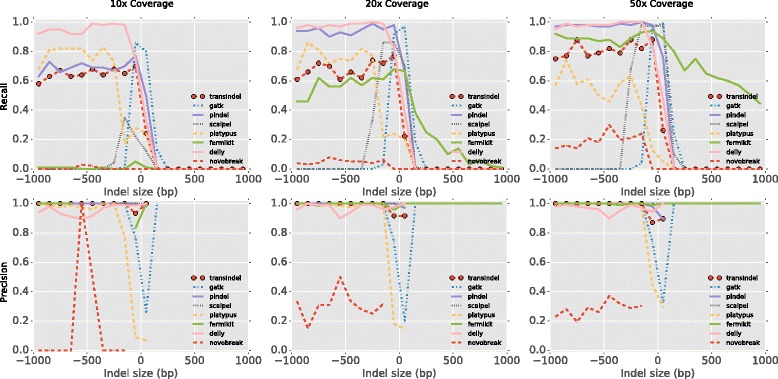
Benchmarking of transIndel for large indels against existing indel detection tools using 100 bp simulated reads. Recall (upper panels) and precision (lower panels) were evaluated for transIndel, GATK HaplotypeCaller, Pindel, Platypus, Scalpel, Delly, FermiKit and NovoBreak. Smoothed histograms (100 bp bins) showed the comparison on simulated data of 10×, 20× and 50× mean coverage for detecting 1000 deletions and 1000 insertions, one each from the size range of 1 bp to 1 kb. Precision was not calculated if a zero denominator was given by the method

We compared the performance of transIndel with seven existing indel detection algorithms (GATK HaplotypeCaller [20], Pindel [21], Scalpel [22], Platypus [23], Fermikit [24], Delly [25] and NovoBreak [26]) on a synthetic data set. This comparison showed that Delly, Pindel and transIndel robustly detected large deletions from low (10×) to high (50×) coverage data with sizes ranging from100bp to 1 kbp (Fig. 2). Fermikit performed the best for detecting large insertions as it is the only one among these tools carrying out global assembly for indel detection. When examining mid-sized and small indels (< 100 bp), Pindel had the highest recall and precision, followed by transIndel (Additional file 3: Figure S2). Delly did not perform as well as Pindel or transIndel in terms of small indels (< 20 bp) (Additional file 3: Figure S2).

**Fig. 3:**
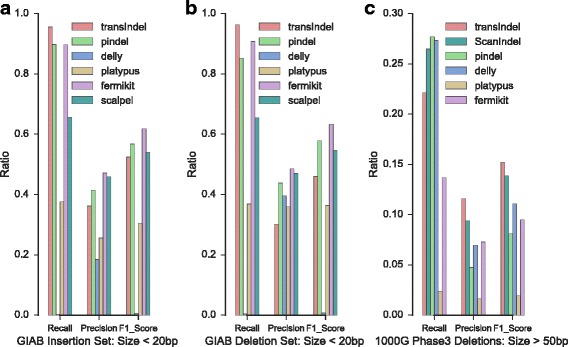
Benchmarking of indels detection using NA12878 whole genome sequencing data. **a** Performance measured by recall, precision and F1 score of small insertions (< 20 bp) detected in Genome in a bottle (GIAB) truth set of small indels in genome NA12878. **b** Performance of small deletions (< 20 bp) detected in GIAB truth set of small indels in genome NA12878. **c** Performance of large deletion (> 50 bp) detection against 1000 Genome Phase 3 deletion call set in genome NA12878

To assess performance with real DNA-seq data, we applied each tool to a 2X100bp, 50× coverage whole genome sequencing (WGS) data set from human individual NA12878. We compared the indel predictions against two reference call sets. One was available from the Genome in a Bottle (GIAB) Consortium, which identified mostly small indels less than 20 bp [27]. The other reference set was composed of mid-sized and large deletions > 50 bp provided by 1000 Genomes Phase 3. We computed the precision, recall (i.e., sensitivity), and harmonic mean of precision and recall (F-measure). We observed that transIndel achieved the highest sensitivity for detecting small indels (Fig. 3a-b). For large deletion detection, transIndel displayed the best performance (measured by F1 score) relative to other structural variation detection algorithms including Delly, Pindel, Platypus, Fermikit as well as our previously developed indel caller, ScanIndel. (Fig. 3c).

Next, we applied transIndel to detect 10 validated large deletions (> 1 kb) within the androgen receptor (AR) gene locus from prostate cancer specimens [14]. TransIndel achieved 100% sensitivity with estimated variant allele frequency (VAF) ranging from 3% to 93% (Additional file 4: Table S1). Notably, we found our calculated VAFs based on SAMTools pileup were lower than the reported VAFs in the original study. For example, a 3433 bp deletion from site A of patient C-6 had a 30% VAF but estimated as 47% by SHEAR originally. Since SAMTools directly used chimeric reads with recovered indels to calculate the VAF which may only account for partial variant depth, it was likely to underestimate the VAF. To make the prediction more accurate, we applied VarDict to predict the VAF of this deletion. VarDict enables accurate estimation of the VAF for indels by performing supervised and unsupervised local realignment of soft-clipped reads [12]. Interestingly, we found VarDict did not detect this deletion using the BWA-MEM generated BAM file (Additional file 5: Figure S3a), but with the redefined BAM file from transIndel, VarDict identified this deletion with 85% VAF, suggesting transIndel improved the sensitivity of existing tools for indel detection (Additional file 5: Figure S3b).

### Application to whole-exome data of primary and metastatic prostate cancer

**Fig. 4:**
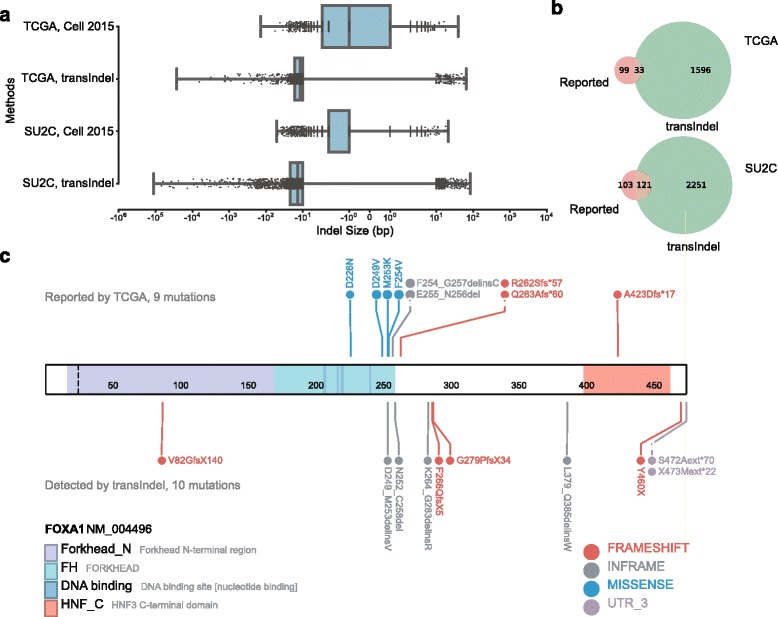
Indel detection on whole exome sequencing (WES) data. **a** The size distribution of detected indels (≥ 10 bp) by transIndel and the reported indels in the TCGA and AACR-PCF-SU2C studies for primary and metastatic castration-resistant prostate cancer, respectively. **b** Venn diagram of medium- and large-sized indels (≥ 10 bp) called by original TCGA and AACR-PCF-SU2C studies versus transIndel. **c** transIndel identified novel deletions in *FOXA1* from ten prostate cancer specimens that were missed by original TCGA study (lower panel)

To test whether transIndel could enhance indel detection in a large dataset relevant to human disease, we first applied transIndel to whole exome sequencing (WES) data of 333 tumor and matched-normal primary prostate cancer (PC) sample pairs from The Cancer Genome Atlas (TCGA) study [15] and 59 metastatic castration-resistant prostate cancer (mCRPC) and matched-normal sample pairs from the AACR-PCF Stand-Up-To-Cancer (SU2C) study [28]. Since existing algorithms can reliably detect SNVs and small indels less than 10 bp (Additional file 3: Figure S2), we focused on indels equal to or larger than 10 bp. In addition, we set the minimal VAF to 10% to keep our approach consistent with thresholds applied in initial analyses of these WES data [15, 28]. We noted that the size of deletions initially called by transIndel varied significantly, with many falling into the structural variation category scale, rather than the indel scale. We therefore further limited our study to indels that were contained within a single gene only. With these filtering thresholds, we detected 1043 somatic indels in PC and 2034 somatic indels in mCRPC. The size range of deletions observed in PC and mCRPC were 10 to 27,293 bp and 10 to 113,612 bp, respectively and the size range of insertions in PC and mCRPC were 10 to 66 bp and 10 to 85 bp, respectively. Compared to the indel detection methods employed in the original TCGA and SU2C studies, transIndel detected more medium- and large-sized indels (Fig. 4a,b). Among these newly detected indels, we found that ten patients in the TCGA cohort harbored deletions in *FOXA1* with sizes larger than 10 bp. *FOXA1* mutations define one of the 7 distinct molecular subtypes of PC, yet nine of these patients had been assigned to a molecular subtype other than *FOXA1* molecular subtype (Additional file 6: Table S2). Re-assignment of the nine patients positive for *FOXA1* indels from the non-*FOXA1* molecular subtype to the *FOXA1* molecular subtype of PC would increase the proportion of this group from 3% to 5% of all PC cases (Fig. 4c).

To ensure we did not miss any additional *FOXA1* indels in the TCGA cohort, we leveraged VarDict to estimate the VAF of our detected indels and also applied Delly, Pindel, Scalpel, GATK, Platypus and Fermikit for detection. We found none of these tools could identify all ten *FOXA1* deletions. Moreover, no additional mid-sized and large indels were found in *FOXA1* by these tools (Additional file 6: Table S2).

### Application to RNA-seq data of mCRPC

**Fig. 5:**
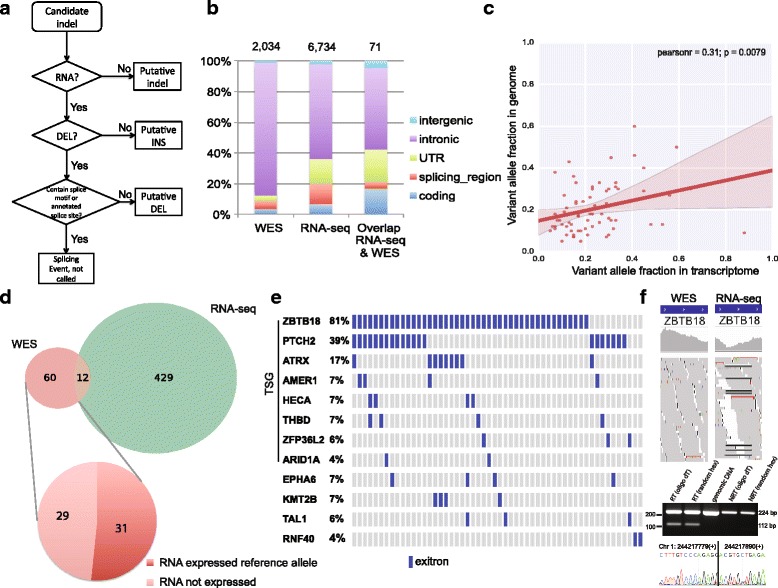
Indel detection on RNA-seq data. **a** transIndel workflow of calling indels from RNA-seq data. **b** The composition of functionally important regions for detected indels within annotated genes in WES and RNA-seq data from AACR-PCF-SU2C samples. **c** Comparison of the indel variant allele fractions derived from RNA-seq and WES data from AACR-PCF-SU2C samples. The line shows a fitted linear regression model with 95% confidence interval. **d** Overlap in coding indels detected from WES and RNA-seq data from AACR-PCF-SU2C samples. Over half of the indels detected in WES data were not detected in RNA-seq data because of no RNA expression, or RNA expression from the un-affected, reference allele. **e** Candidate recurrent exitron splicing events in tumor suppressor genes (TGS) and other cancer-related genes in metastatic castration-resistant prostate cancer. **f** Validation of a 112 bp *ZBTB18* exitron splicing event in RNA but not DNA from the LNCaP cell line. Genomic coordinates are hg19. RT = reverse transcriptase, NRT = no reverse transcriptase. Reverse transcription reactions were primed with oligo-dT primers or random hexamers as indicated

To validate our findings from WES data and evaluate effects of detected genomic indels on transcriptome structure, we applied transIndel to RNA-seq data from 59 mCRPC samples in the SU2C cohort. RNA-seq raw reads were aligned with BWA-MEM and transIndel was used to infer candidate indels. Deletion candidates were subjected to several filters to discriminate genomic deletions from RNA splicing events, including removal of deletions neighboring canonical splicing motifs or annotated splice sites (Fig. 5a). This pipeline identified genomic deletions that were misclassified as splicing junctions (Additional file 7: Figure S4a-d) and genomic insertions that were missed (Additional file 7: Figure S4e-h) by the STAR algorithm, which is a component of GATK best practice for indel discovery in RNA-seq (https://software.broadinstitute.org/gatk/best-practices/rnaseq.php). Compared to WES, transIndel called more indels from RNA-seq data, and these were more abundant in untranslated regions (UTRs), splicing regions, and coding exonic regions (Fig. 5b). This situation likely occurred because the exome capture kits used for WES are primarily designed to capture the protein-coding regions in the genome (~ 2% of human genome), whereas RNA-seq also covers UTRs and retained introns in the transcriptome (~ 5% of human genome) [29]. Indeed, we found the largest proportion of detected indels from RNA-seq data were intronic indels (Additional file 8: Figure S5).

A surprising result was that WES indel calls overlapped with indels discovered in RNA-seq to a very limited extent (Fig. 5b). To investigate the reasons for this low degree of overlap, we examined differences in WES and RNA-seq read coverage at locations corresponding to the 2034 WES indels (Additional file 9: Figure S6a) and 6734 RNA-seq indels (Additional file 9: Figure S6b). These results indicated that coverage difference between WES and RNA-seq at the locations of predicted indels was the main reason for this low overlap in indel calls.

As anticipated, the fraction of indels in coding exons was markedly higher in the overlap between WES and RNA-seq than in either dataset alone (Fig. 5b). Interestingly, the VAFs of the 71 common indels called from WES and RNA-seq data showed weak correlation (*r* = 0.31;*p* = 0.0079). Linear regression analysis indicated that indels called from RNA-seq data were present at higher VAF than those same indels called from WES data (Fig. 5c). This is likely due to inefficient hybridization-based capture of DNA fragments harboring medium- to large-scale indels, which is a standard step in WES workflows. An example of this scenario is shown for *FOXA1* in Figure S7 (Additional file 10). We found the WES didn’t cover FOXA1 well between aa240–380, which might prevent detecting additional FOXA1 mutations**.** These results suggest that detection of indels in RNA-seq data using transIndel could complement WES for detection and validation of genomic alterations, which is crucial for clinical diagnostics.

Since WES and RNA-seq data are both expected to display enrichment in exonic regions, we focused on the indels discovered in coding exons. Surprisingly, we found only 12 coding indels called from WES data that overlapped with coding indels called from RNA-seq data (Fig. 5d). Explanations for this surprisingly low overlap could be that the genes impacted by indels were not expressed (RPKM < 1), or gene expression was arising from the unaffected, reference allele (Fig. 5d). Overall, there were more coding indels called from RNA-seq data. In several instances, manual inspection of WES data indicated the presence of individual DNA-seq reads supporting these indel events. However, these would have ultimately been filtered out in the DNA-seq indel calling pipeline due to low coverage (< 10×). Conversely, many coding indels called from RNA-seq data appeared to be RNA-only events, as there was no evidence of corresponding DNA-levels events despite high WES coverage in these regions. One explanation for this phenomenon could be a type of non-canonical splicing event, termed exonic introns (exitron) [30]. One of the distinguishing features of exitrons is overrepresentation of indel sizes that are multiples of three nucleotides [31]. Indeed, by comparing the size distribution of detected exitron events with all RNA-seq indels, we found that exitron events were enriched for indels with sizes that were multiples of three (Additional file 11: Figure S8).

Although the transIndel pipeline for indel detection from RNA-seq data included the removal of deletions that could be splicing junctions, the catalog of alternative splicing events in prostate cancer, including exitrons, is incomplete. Remarkably, many of the detected exitrons were recurrent within genes known to be altered at high frequency in cancer (Fig. 5e). Over half of the cancer genes displaying non-canonical exitron splicing were known tumor suppressor genes (TSGs). We also found that *KMT2B*, a known regulator of mCRPC [32], displayed exitron splicing in 7% of mCRPC samples. To test the accuracy of these RNA-level calls, we assessed their presence in the LNCaP cell line by analyzing LNCaP WES and RNA-seq data using transIndel. LNCaP cells displayed evidence for exitron splicing in *ZBTB18*, which was the most frequent exitron splicing events observed in mCRPC specimens. We performed RT-PCR with LNCaP RNA and PCR with LNCaP DNA, and confirmed by Sanger sequencing that *ZBTB18* displayed bona fide exitron splicing at the RNA level only (Fig. 5f). *ZBTB18* encodes a transcriptional repressor involved in neural development. It has been reported as a possible tumor suppressor as its expression reduces cell proliferation [33]. Additionally, it has been reported that the *ZBTB18* exitron splicing expressed in both *ERBB2*-positive breast cancer and normal breast tissues, but it has higher percent of spliced in value in tumor compared with normal samples [30].

## Discussion

Herein, we have devised transIndel, a computational approach that allows the accurate identification of indels from DNA-seq or RNA-seq. TransIndel is able to call small indels from normal alignments and reconstruct large deletions and mid-sized insertions from chimeric alignments. When applied to RNA-seq data, transIndel carries out subsequent filtering that accounts for annotated RNA splicing events. By analyzing whole exome DNA-seq data in TCGA and SU2C cohorts, we demonstrated that transIndel is highly sensitive for discovering mid-sized and large indels that were missed by these original studies. Applying transIndel to RNA-seq data allowed us to validate expression of indels detected from DNA-seq and discover large indels that were missed by DNA-seq due to inefficient capture of DNA fragments harboring these indels.

One major improvement of transIndel compared with other indel callers is the ability to infer indels directly from the initial alignment given by the short read aligner. Most of the existing indel detect methods, such as Pindel [21] and ScanIndel [13], rely on an ad hoc step for realigning split reads to determine whether those reads support an indel event or not. These realignment steps are carried out either by a third-party aligner (e.g. BLAT used by ScanIndel) or internal realignment algorithms (e.g. pattern growth algorithms used by Pindel). Although these realignment steps can help identify indels that are not marked by the initial alignment, they significantly increase the running time of those tools. We realize that although there is not hard evidence for the existence of indels within Compact Idiosyncratic Gapped Alignment Report (CIGAR) strings, chimeric alignments provide sufficient information to directly identify an indel event by comparing the read length and the target offset calculated from the split linear alignments in the chimeric alignment. Our benchmarks using synthetic and real data have demonstrated that transIndel exhibited competitive performance with the tools using realignment strategies for mid-sized and large indel detection. The major advance achieved by eliminating this realignment step is that transIndel can analyze RNA-seq alignment data, which possess enormous numbers of split reads due to splicing, in a reasonable running time.

RNA-seq data has been used traditionally for measuring gene expression levels, and identifying novel splicing isoforms, non-coding RNAs, and gene fusions [34]. Recently, RNA-seq data has been utilized to validate the expression of single nucleotide variants (SNVs) identified from DNA-seq data [2, 4]. To our knowledge, transIndel is the first algorithm to enable evaluation of expressed mid-sized and large indels in RNA-seq data. We found that inclusion of RNA-seq data in indel discovery strategies greatly increased sensitivity for detecting indels in prostate cancer. This work complements a previous report focusing on detection of point mutations and small indels, which also found that analysis of RNA-data enhanced discovery of these variants over analysis of DNA-seq data alone [4].

Currently, existing tools for detection of SNVs from RNA-seq data require integrated analysis of DNA-seq data with the intent of balancing sensitivity and specificity [2, 4]. TransIndel is capable of detecting indels from RNA-seq alone. This is expected to greatly enhance the usability of RNA-seq data, as transIndel would enable analysis of the large numbers of samples that have been analyzed by RNA-seq for which matched DNA-seq data are not available. However, one caveat noted in our study is that transIndel annotates non-canonical exitron splicing as deletions if DNA-seq is not available for validation.

Overall, our work demonstrates the feasibility of indel calling from RNA-seq data with high sensitivity and specificity. Recently, it has become more common for clinical testing pipelines to employ both whole exome DNA-seq and RNA-seq analyses. We anticipate that transIndel will serve as a powerful tool that will empower the exploration of genomic indels from both DNA-seq and RNA-seq data.

## Conclusions

Our study demonstrates that transIndel is a robust tool for elucidation of medium- and large-sized indels from DNA-seq and RNA-seq data. Including RNA-seq data in indel discovery efforts leads to significant improvements in sensitivity for identification of indels missed by WES, and reveals non-canonical RNA-splicing events in genes associated with disease pathology.

## Availability and requirements

**Project name:** TransIndel.

**Project home page:** https://github.com/cauyrd/transIndel

**Operating system(s):** Platform independent.

**Programming language:** Python.

**Other requirements:** N/A.

**License:** The GNU General Public License (GPL).

**Any restrictions to use by non-academics:** N/A.

## Additional files

Additional file 1: **Dataset S1.** List of cancer related genes. (XLSX 92 kb)

Additional file 2: **Figure S1.** Distribution of different type of aligned reads at various deletion junctions by STAR and BWA-MEM. (PDF 70 kb)

Additional file 3: **Figure S2.** Benchmarking of transIndel for mid-sized indels against existing indel detection tools using 100 bp simulated reads. (PDF 221 kb)

Additional file 4: **Table S1.** Summary of deletions in AR detected by transIndel. (XLSX 37 kb)

Additional file 5: **Figure S3.** TransIndel detects a 3433 bp deletion in AR from human prostate cancer sample. (PDF 330 kb)

Additional file 6: **Table S2.** FoxA1 deletion detection by different methods from TCGA whole exome sequencing data. (XLSX 40 kb)

Additional file 7: **Figure S4.** Examples of detected 214 bp deletion in 3’UTR of *ATAD5* and 30 bp insertion in exon 15 of *EPS15* by exome-seq and RNA-seq in SU2C samples. (PDF 85 kb)

Additional file 8: **Figure S5.** RNA-seq coverage for annotated exons and introns in subject 1,115,156 from SU2C cohort. (PDF 52 kb)

Additional file 9: **Figure S6.** Sequencing coverage comparison of detected WES and RNA-seq indels in SU2C cohort. (PDF 97 kb)

Additional file 10: **Figure S7.** An example of *FOXA1* deletion detected by RNA-seq but missed by WES due to low coverage in DNA sequencing. (PDF 56 kb)

Additional file 11: **Figure S8.** Size distribution of identified exitrons and all RNA-seq indels from SU2C samples. (PDF 37 kb)

## Abbreviations

bp: base pair
CCLE: Cancer Cell Line Encyclopedia
CIGAR: Compact Idiosyncratic Gapped Alignment Report
Indel: Insertion and Deletion
mCRPC: metastatic castration-resistant prostate cancer (mCRPC)
PC: Primary prostate cancer
SU2C: Stand Up To Cancer
TCGA: The Cancer Genome Atlas
WES: Whole exome sequencing
WGS: Whole genome sequencing
