# Supplementary material for: Indel detection from DNA and RNA sequencing data with transIndel
Source: BMC Genomics. 2018 Apr 19;19:270. doi: 10.1186/s12864-018-4671-4 (PMC5909256; doi:10.1186/s12864-018-4671-4)
Supplement: Supplementary file 2 — Figure S1. Distribution of different type of aligned reads at various deletion junctions by STAR and BWA-MEM. (PDF 70 kb) [file 12864_2018_4671_MOESM2_ESM.pdf]

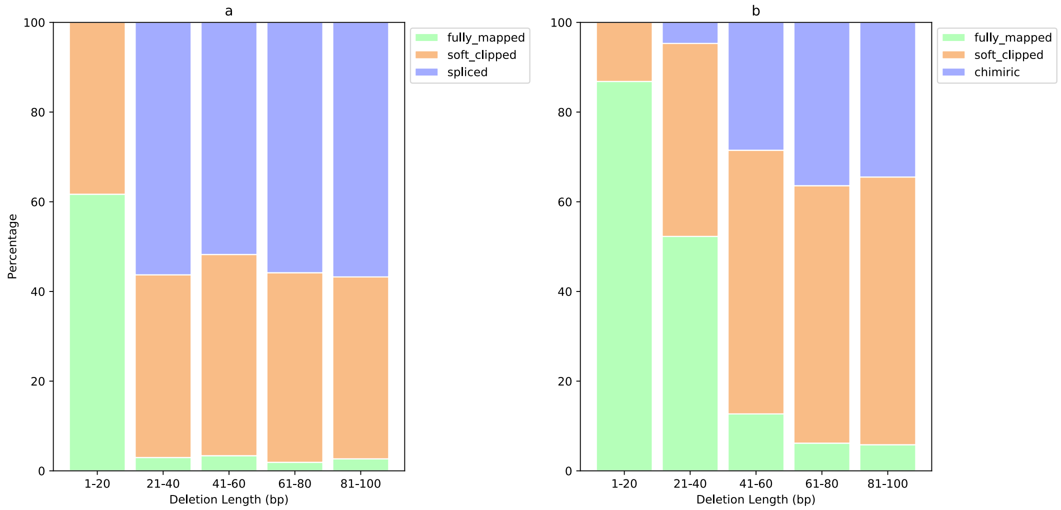

Figure S1: Distribution of different type of aligned reads at various deletion junctions by STAR and BWA-MEM. Simulated 100bp pair-end reads were generated to sequence 50x coverage of modified human chromosome 20 with added deletion event of length 1-100bp. The reads around the deletions aligned by STAR **(a)** fall into three types: fully mapped reads, splicing junction reads and soft-clipped reads. The reads around the deletions aligned by BWA-MEM **(b)** fall into fully mapped reads, chimeric reads and other soft-clipped reads. The distribution of different group of reads generated by the two aligners are counted against various deletion length.
