# Supplementary material for: Indel detection from DNA and RNA sequencing data with transIndel
Source: BMC Genomics. 2018 Apr 19;19:270. doi: 10.1186/s12864-018-4671-4 (PMC5909256; doi:10.1186/s12864-018-4671-4)
Supplement: Supplementary file 3 — Figure S2. Benchmarking of transIndel for mid-sized indels against existing indel detection tools using 100 bp simulated reads. (PDF 221 kb) [file 12864_2018_4671_MOESM3_ESM.pdf]

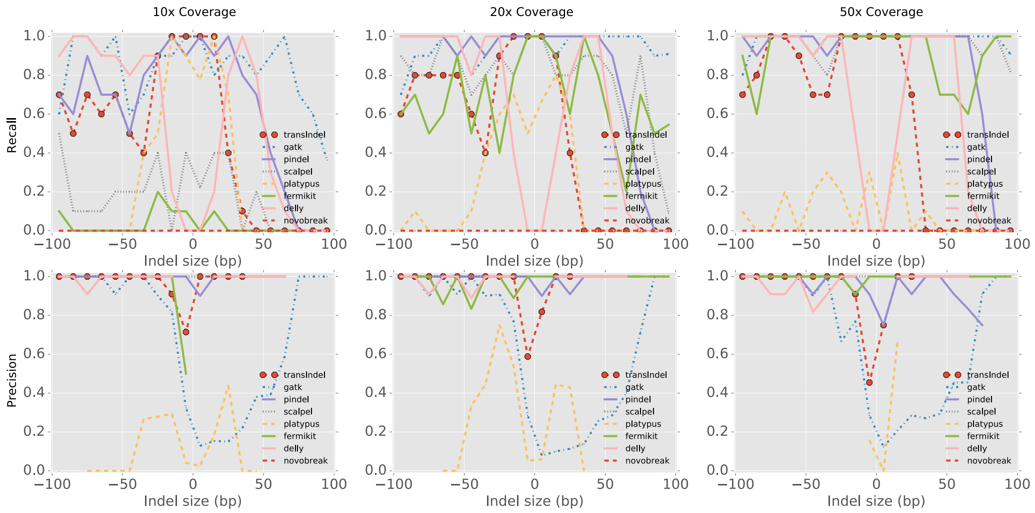

Figure S2: Benchmarking of transIndel for mid-sized indels against existing indel detection tools using 100bp simulated reads. Recall (upper panels) and precision (lower panels) were evaluated for transIndel, GATK HaplotypeCaller, Pindel, Platypus, Scalpel, Delly, FermiKit and NovoBreak. Smoothed histograms (10bp bins) show comparisons with simulated data at 10x, 20x and 50x mean coverage for detecting 100 deletions and 100 insertions, one each from the size range of 1bp to 100bp. Precision was not calculated if a zero denominator was given by the method.
