# Supplementary material for: Indel detection from DNA and RNA sequencing data with transIndel
Source: BMC Genomics. 2018 Apr 19;19:270. doi: 10.1186/s12864-018-4671-4 (PMC5909256; doi:10.1186/s12864-018-4671-4)
Supplement: Supplementary file 5 — Figure S3. TransIndel detects a 3433 bp deletion in AR from human prostate cancer sample. (PDF 330 kb) [file 12864_2018_4671_MOESM5_ESM.pdf]

a

b

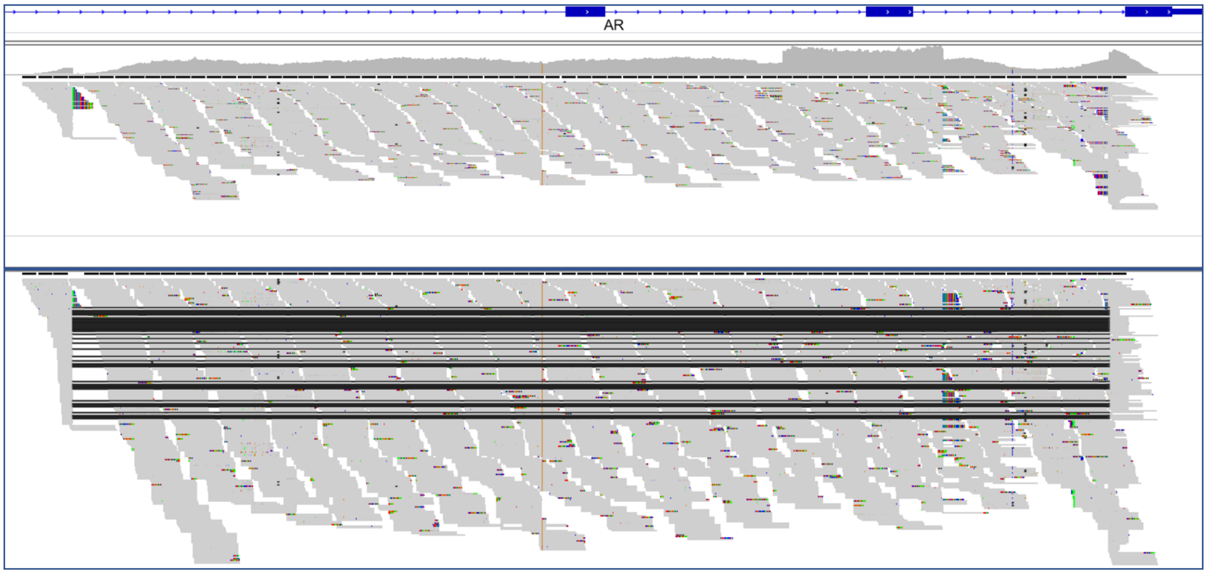

Figure S3: TransIndel detects a 3,433bp deletion in AR from human prostate cancer sample. The breakpoints of the deletions are marked by soft-clipped reads and when applying VarDict (with –I parameter set to 4000) to detect this deletion, it failed to identify this deletion **(a).** TransIndel reconstruct the deletions from the chimiric reads marked by the black lines in **(b)** and VarDict successfully detect this deletion using transIndel's output BAM file with a predicted 85% VAF.
