# Supplementary material for: Indel detection from DNA and RNA sequencing data with transIndel
Source: BMC Genomics. 2018 Apr 19;19:270. doi: 10.1186/s12864-018-4671-4 (PMC5909256; doi:10.1186/s12864-018-4671-4)
Supplement: Supplementary file 7 — Figure S4. Examples of detected 214 bp deletion in 3’UTR of ATAD5 and 30 bp insertion in exon 15 of EPS15 by exome-seq and RNA-seq in SU2C samples. (PDF 85 kb) [file 12864_2018_4671_MOESM7_ESM.pdf]

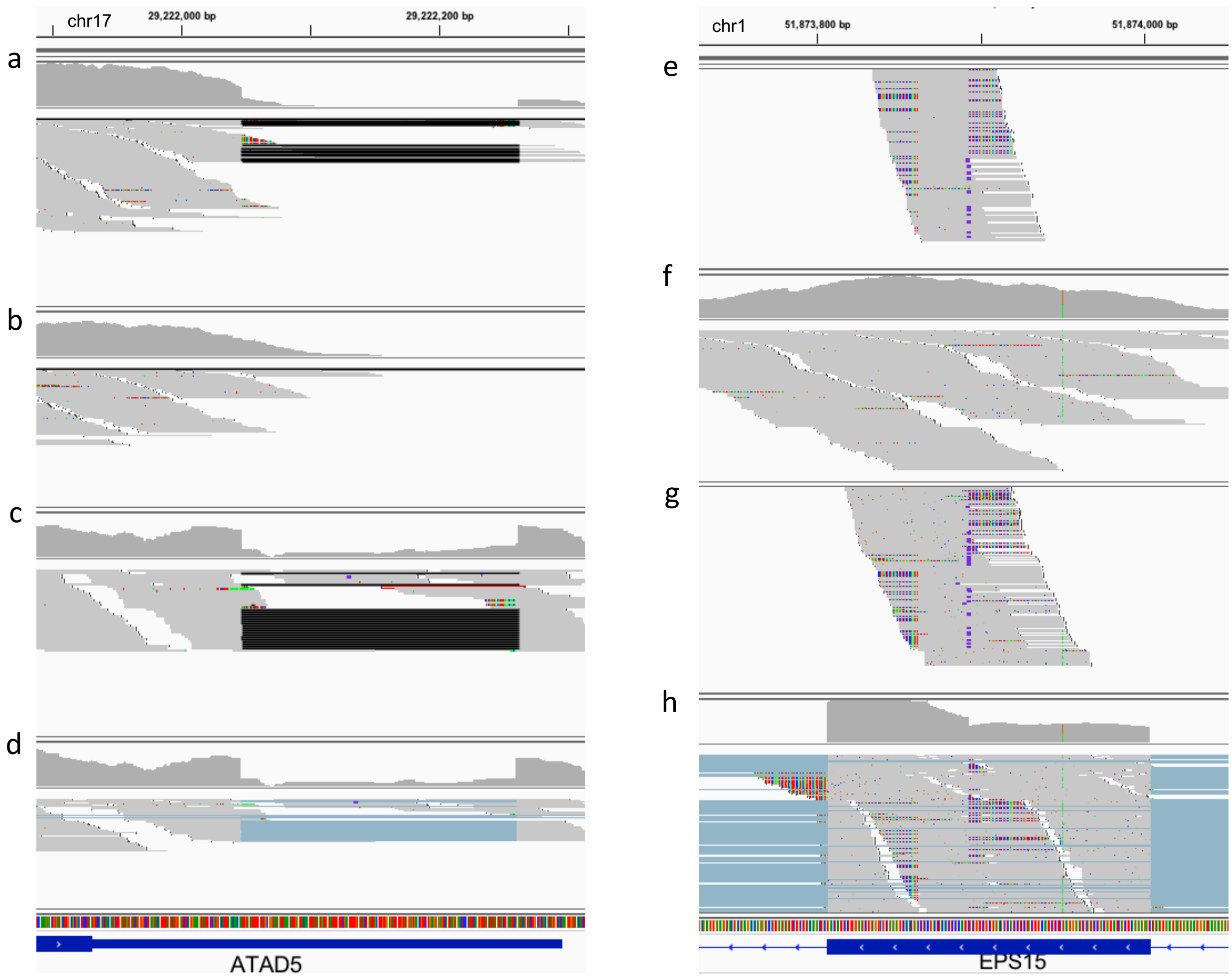

Figure S4: Examples of detected 214bp deletion in 3'UTR of *ATAD5* and 30bp insertion in exon 15 of *EPS15* by exome-seq and RNA-seq in SU2C samples. (a) A 214bp deletion of *ATAD5* (black lines) detected in DNA-seq data by transIndel in SU2C subject MO_1176. **(b)** The exome sequencing data from matched normal tissues. **(c)** The same deletion detected in (a) by transIndel using RNA-seq data from this subject. **(d)** This deletion was called as a splicing event (blue lines) when RNA-seq data was analyzed by the STAR algorithm. **(e)** A 30bp insertion (purple bars) in *EPS15* detected in DNA-seq data by transIndel in SU2C subject 1115183. **(f)** The exome sequencing data from matched normal tissues. **(g)** The same insertion detected in (d) by transIndel using RNA-seq data from this subject. **(h)** This insertion was not detected by the STAR algorithm (Genomic coordinate is hg19).
