# Supplementary material for: Indel detection from DNA and RNA sequencing data with transIndel
Source: BMC Genomics. 2018 Apr 19;19:270. doi: 10.1186/s12864-018-4671-4 (PMC5909256; doi:10.1186/s12864-018-4671-4)
Supplement: Supplementary file 8 — Figure S5. RNA-seq coverage for annotated exons and introns in subject 1,115,156 from SU2C cohort. (PDF 52 kb) [file 12864_2018_4671_MOESM8_ESM.pdf]

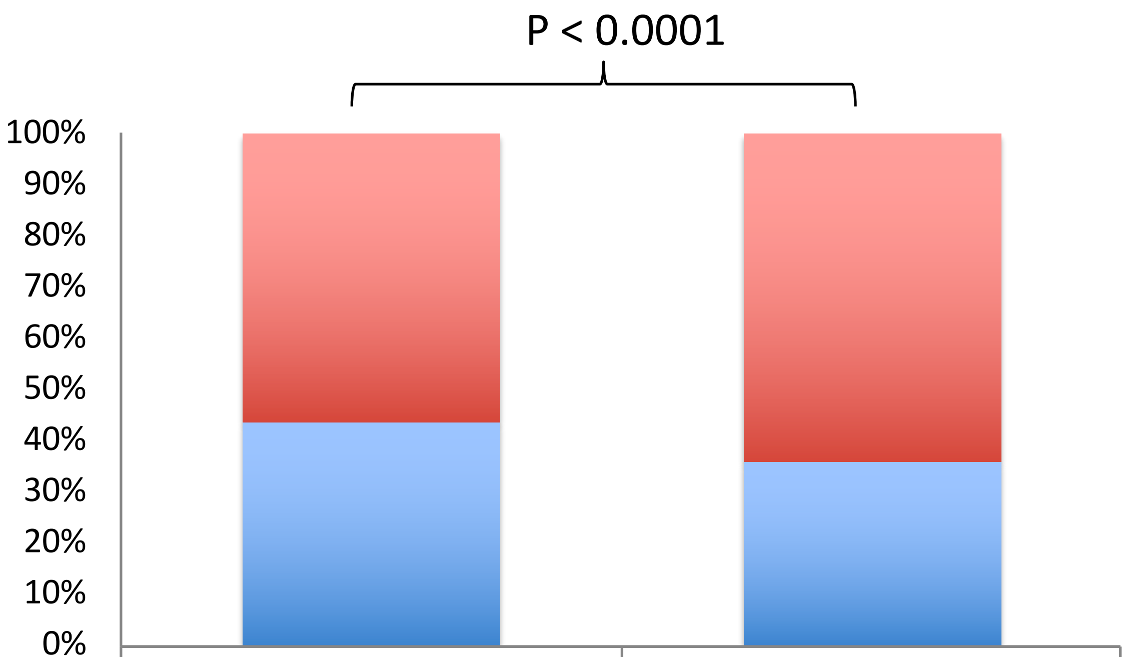

	Exon	Intron
Covered	111123	116303
No Read	86055	64602

Figure S5: RNA-seq coverage for annotated exons and introns in subject 1115156 from SU2C cohort. The hg19 genomic coordinates of RefSeq coding exons and introns were obtained from UCSC genome browser. GATK DiagnoseTargets tool was used to calculate the coverage distribution for exon and intron regions using the RNA-seq data of the subject 115156. The P-value is calculated using Fisher's exact test.
