# Supplementary material for: Indel detection from DNA and RNA sequencing data with transIndel
Source: BMC Genomics. 2018 Apr 19;19:270. doi: 10.1186/s12864-018-4671-4 (PMC5909256; doi:10.1186/s12864-018-4671-4)
Supplement: Supplementary file 9 — Figure S6. Sequencing coverage comparison of detected WES and RNA-seq indels in SU2C cohort. (PDF 97 kb) [file 12864_2018_4671_MOESM9_ESM.pdf]

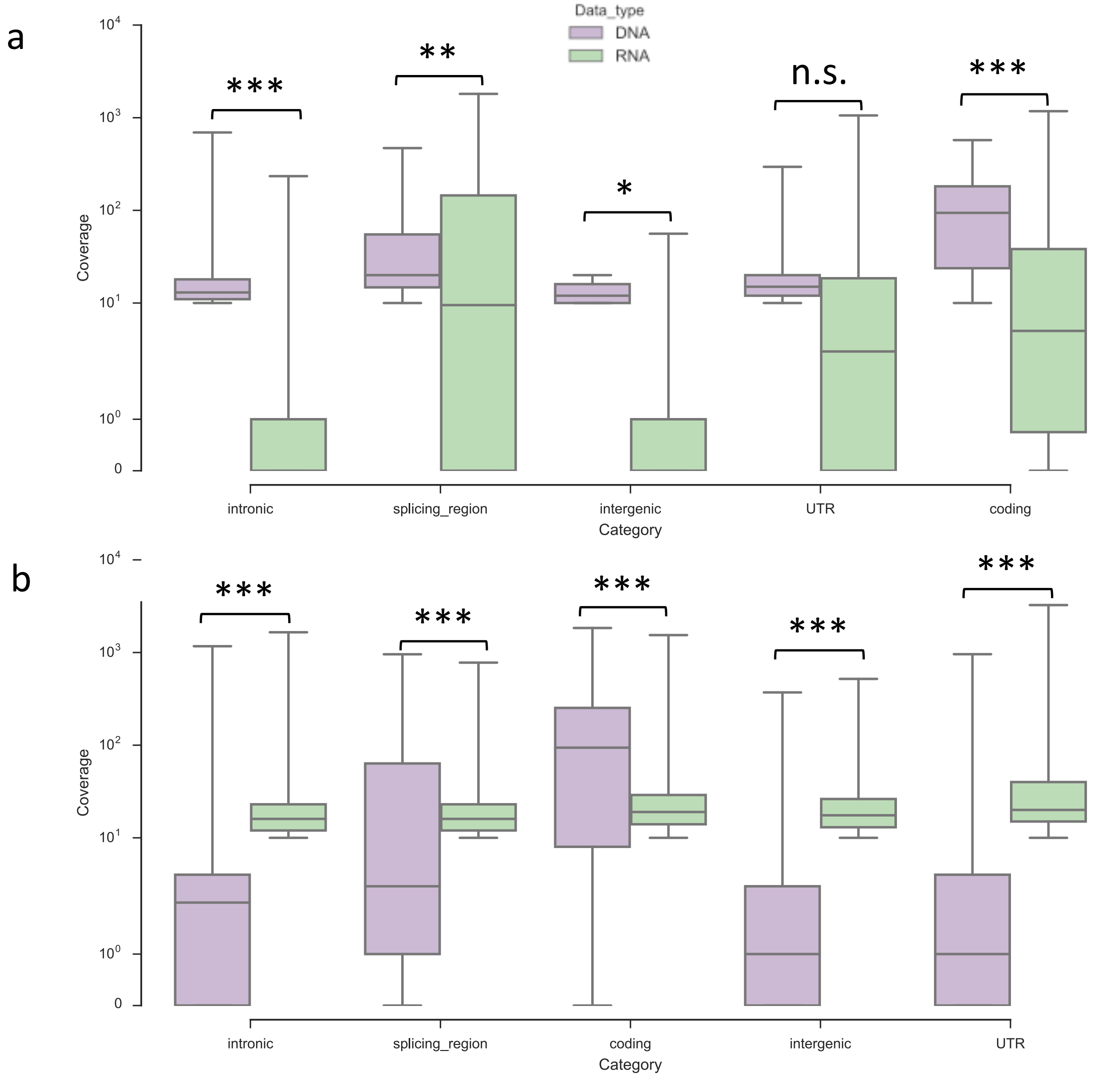

Figure S6: Sequencing coverage comparison of detected WES and RNA-seq indels in SU2C cohort. Sequence coverage of DNA-seq and RNA-seq data at locations corresponding to 2,034 predicted WES indels (a) and 6,734 predicted RNA-seq indels (b). Indels detected from WES and RNA-seq were counted across intergenic, coding, intronic, UTR, and splicing regions as indicated. P-values were calculated using student t-test: *P≤0.05, **P≤0.01, ***P≤0.001, n.s., not significant.
