# Supplementary material for: Indel detection from DNA and RNA sequencing data with transIndel
Source: BMC Genomics. 2018 Apr 19;19:270. doi: 10.1186/s12864-018-4671-4 (PMC5909256; doi:10.1186/s12864-018-4671-4)
Supplement: Supplementary file 10 — Figure S7. An example of FOXA1 deletion detected by RNA-seq but missed by WES due to low coverage in DNA sequencing. (PDF 56 kb) [file 12864_2018_4671_MOESM10_ESM.pdf]

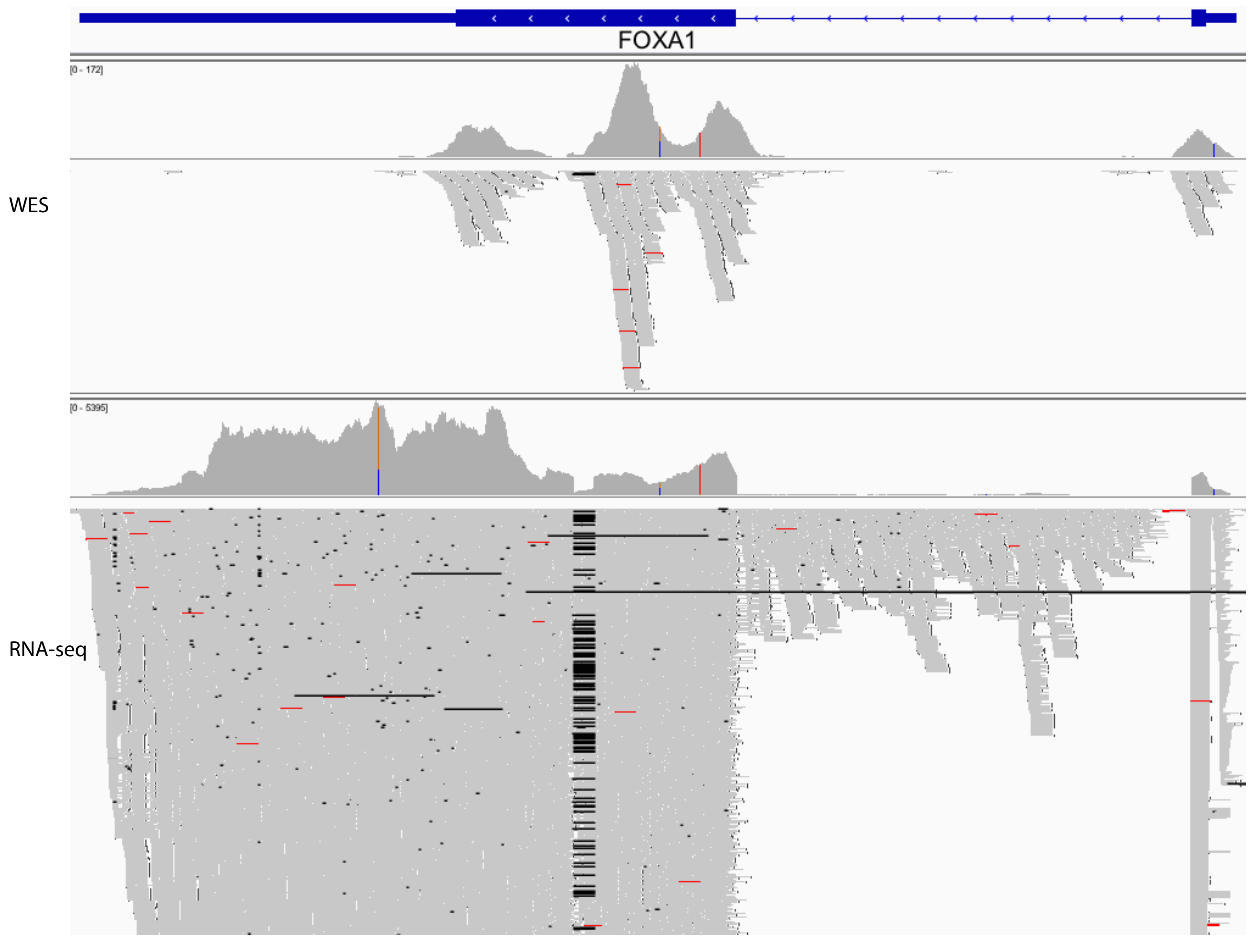

Figure S7: An example of *FOXA1* deletion detected by RNA-seq but missed by WES due to low coverage in DNA sequencing. A 95bp deletion is detected in exon 2 of *FOXA1* in SU2C subject 1115161 at chr14:38,061,136-38,061,230 (hg19 coordinate) by RNA-seq with 33% variant allele fraction. This deleted region is covered by only 9 reads in whole exome DNA-seq data.
