# Supplementary material for: Indel detection from DNA and RNA sequencing data with transIndel
Source: BMC Genomics. 2018 Apr 19;19:270. doi: 10.1186/s12864-018-4671-4 (PMC5909256; doi:10.1186/s12864-018-4671-4)
Supplement: Supplementary file 11 — Figure S8. Size distribution of identified exitrons and all RNA-seq indels from SU2C samples. (PDF 37 kb) [file 12864_2018_4671_MOESM11_ESM.pdf]

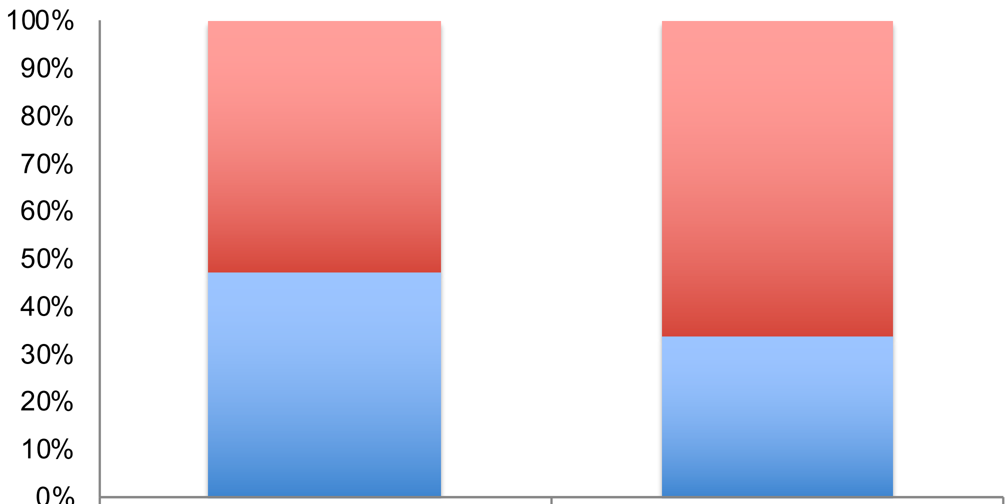

	Exitron	All RNA-seq indel
Not multiple of three	123	4440
Multiple of three	110	2294

Figure S8: Size distribution of identified exitrons and all RNA-seq indels from SU2C samples. Size of detected exitrons and indels from RNA-seq data in SU2C cohort, represented by whether or not they were multiples of three nucleotides. Fisher's exact test was used to determine whether exitron events were enriched for indels with sizes that were multiples of three.
